# Integrated Process Modeling—A Process Validation Life Cycle Companion

**DOI:** 10.3390/bioengineering4040086

**Published:** 2017-10-17

**Authors:** Thomas Zahel, Stefan Hauer, Eric M. Mueller, Patrick Murphy, Sandra Abad, Elena Vasilieva, Daniel Maurer, Cécile Brocard, Daniela Reinisch, Patrick Sagmeister, Christoph Herwig

**Affiliations:** 1 Exputec GmbH, Mariahilferstraße 147, 1150 Vienna, Austria; thomas.zahel@exputec.com (T.Z.); stefan.f.hauer@gmx.at (S.H.); patrick.sagmeister@exputec.com (P.S.); 2 Versartis Inc., 4200 Bohannon Drive, Suite 250, Menlo Park, CA 94025, USA; emueller@versartis.com (E.M.M.); pmurphy@versartis.com (P.M.); 3 Boehringer Ingelheim RCV GmbH & Co KG, Doktor-Boehringer-Gasse 5-11, 1120 Vienna, Austria; sandra.abad@boehringer-ingelheim.com (S.A.); elena.vasilieva@boehringer-ingelheim.com (E.V.); daniel.maurer@boehringer-ingelheim.com (D.M.); cecile.brocard@boehringer-ingelheim.com (C.B.); daniela.reinisch@boehringer-ingelheim.com (D.R.)

**Keywords:** process validation, process characterization study, holistic process model, predict out of specification events, Monte Carlo simulation, biopharmaceutical manufacturing

## Abstract

During the regulatory requested process validation of pharmaceutical manufacturing processes, companies aim to identify, control, and continuously monitor process variation and its impact on critical quality attributes (CQAs) of the final product. It is difficult to directly connect the impact of single process parameters (PPs) to final product CQAs, especially in biopharmaceutical process development and production, where multiple unit operations are stacked together and interact with each other. Therefore, we want to present the application of Monte Carlo (MC) simulation using an integrated process model (IPM) that enables estimation of process capability even in early stages of process validation. Once the IPM is established, its capability in risk and criticality assessment is furthermore demonstrated. IPMs can be used to enable holistic production control strategies that take interactions of process parameters of multiple unit operations into account. Moreover, IPMs can be trained with development data, refined with qualification runs, and maintained with routine manufacturing data which underlines the lifecycle concept. These applications will be shown by means of a process characterization study recently conducted at a world-leading contract manufacturing organization (CMO). The new IPM methodology therefore allows anticipation of out of specification (OOS) events, identify critical process parameters, and take risk-based decisions on counteractions that increase process robustness and decrease the likelihood of OOS events.

## 1. Introduction

The main goal of pharmaceutical manufacturing is to constantly deliver high product quality, which is reflected in regulatory guidelines [1,2,3]. Process validation is a major initiative to demonstrate the capability of meeting this goal and is separated in three stages (stage 1 to 3). Stage 1 aims at establishing a process design in which process variation in critical quality attributes (CQAs) is understood and connected to critical process parameters. This is usually done within a process characterization study using design of experiment (DoE) strategies. Resulting critical process parameters that have an effect on product quality require sufficient control strategies. Stage 2 consists of process performance qualification (PPQ) runs to confirm the design of the process and ensure it can consistently deliver high product quality. Stage 3, continued process verification (CPV), is an ongoing evaluation and monitoring to confirm the process remains in a state of control or to identify if new interdependencies between process parameters (PPs) and CQAs arise. Those three stages can be seen interlinked to each other as a lifecycle, where potential changes and associated risk in PPQ or routine manufacturing must be iteratively evaluated together with knowledge gained from initial process design [4]. Insufficient risk estimation of the entire process at stage 1 of process design (e.g., in terms of estimation of out of specification events) can lead to inconsistent or unpredicted performance at later stages.

Risk evaluation of individual unit operation of a pharmaceutical processes is commonly conducted by following steps in accordance with ICH Q8 guideline [2]:

- Risk assessment using knowledge of process experts, which leads to a candidate set of potential critical PPs for each unit operation.
- Experimental investigation of the impact of potentially critical PPs onto CQAs. This is usually performed in DoE approaches and statistical regression modeling is used to describe the relationship between significantly impacting critical PPs and CQAs mathematically.
- Comparison of the output of statistical model predictions within normal operating ranges or a design space to pre-defined acceptance limits for each unit operation.
- The risk of not meeting acceptance limits is mitigated by applying an appropriate control strategy, such as a reduction of the normal operating range.

One difficulty, especially in biopharmaceutical manufacturing where multiple unit operations are stacked together and critical PPs interact, is an appropriate evaluation of risk related to impurities. Risk analysis is impeded since propagation of impurities is rarely assessed holistically but rather evaluated on each unit operation separately [5]. Impurity propagation through multiple unit operations is difficult to study with reasonable representative experimental effort, especially at early stages of process design where only a limited number of manufacturing runs is available. However, simulations and modeling are necessary and useful to assess the chance of out of specification events. Having such a predictive tool in place to develop robust processes by incorporating knowledge acquired during process development and characterization experiments, unexplained variance in product quality possibly leading to recalls, complaints, and patient risk can be reduced. Therefore, it is desirable to formulate holistic process and production control strategies that prevent out of specification (OOS) events which could have already been anticipated within the design phase [6]. However, to the best of our knowledge, it has not been shown so far how a holistic risk evaluation spanning over multiple unit operation can be performed at process validation stage 1 and used to demonstrate overall process capability.

MC simulation is a tool to incorporate random variability into the modeled system and connect single modeling-units together. A random sampling distribution for the model parameters (inputs) needs be defined a priori, which does not need to be necessarily normally distributed. Within each cycle of the MC simulation, a different random set of inputs is drawn leading to discrete model results (outputs). Since a large number of MC cycles are performed, it is possible to aggregate the discrete model outputs to a predictive distribution of those outputs. Using this distributional information, it is possible to calculate probabilities of events (e.g., OOS). MC simulations have shown great potential in pharmaceutical industry for drug discovery and simulation of clinical trials [7] and is also routinely utilized for error propagation [8]. However, it has, to our knowledge, not been applied to impurity propagation of a batch-wise pharmaceutical processes.

Herein, we describe the development of an integrated process model (IPM) that is capable of capturing development and design data from multiple unit operations and is able to predict the risk of OOS probabilities through Monte Carlo simulation even at the early design stage of process validation. Moreover, we identify how variance and changes in set point of process parameters impacts drug substance quality. The model can be enriched at later stages also with data from PPQ, routine manufacturing, or additional development. Thereby, a continuous process data management is enabled and risk-based decision making during change and deviation management in continuous manufacturing can be based on the full spectrum of development, design, and manufacturing data.

At this stage, the following derived acceptance criteria for the IPM can be formulated:

- Prove process robustness of an existing design space: Prove that under normal manufacturing conditions it is unlikely to miss drug substance specification for defined CQAs
- Test process robustness under accelerated variance of process parameters and increased impurity burden
- Establish a platform that leverages process knowledge from PV stage 1 for further usage within PPQ and CPV (Stage 2 and 3 of process validation)

With this contribution we present the development of an IPM, validate the IPM using large scale manufacturing data, and demonstrate the capability of the IPM in estimating OOS probabilities under normal and accelerated conditions. This case study was recently conducted at a leading biopharmaceutical CMO in contract development of a novel long acting human growth hormone product.

## 2. Materials and Methods

Here we want to summarize the required inputs for the IPM as well as their assumptions that must be met in order to ensure reliable prediction of the IPM (for details see referred sections):

- Description of the process, order of unit operations, and variance of PPs under normal operating conditions (see Section 2.1). It is assumed that estimation of variance of PPs is representative for routine manufacturing.
- Optional: If initial unit operation of the process is not modeled by the IPM the starting distribution of each CQA needs to be estimated at the starting unit operation of the IPM. It is assumed that the estimation of starting distribution is representative for the real CQA distribution under routine manufacturing (see Section 2.2).
- Statistical regression models that describe significant relationships between PPs and CQAs for each unit operation (see Section 2.3.1). It is assumed that scientifically sound analytical methods (high accuracy, precision, robustness, selectivity, etc.) have been used to record the data that led to formation of those regression models. Moreover, it is assumed that no critical effect has been overlooked, which can be tested using power analysis approaches [9]. This ensures that residual variance in the regression models can be attributed to normal analytical- and process variance.
- Optional: Statistical spiking models of each unit operation describing the dependency between varied impurity load and specific impurity clearance (see Section 2.3.2). Identical assumptions as for the regression models must be met.

### 2.1. Description of Biopharmaceutical Manufacturing Process

This industrial biopharmaceutical process produces a pharmaceutically active recombinant protein and is divided into 7 unit operations. After fermentation using *Escherichia coli* as host cells and recombinant expression of the product, a cell lysis step is performed prior to a precipitation step and clarification. After these primary recovery steps, three preparative chromatographic columns are performed to clear the product from impurities. A final ultrafiltration/diafiltration is performed to adjust the product concentration in drug substance. Two process-related impurities as well as 2 product-related impurities were defined as the major CQAs and herein are modeled within the IPM. Since the analytical quantification of those CQAs was only possible in the load of the first chromatographic step, this step was set as input to the IPM. A summary of the relevant unit operations for modeling, their varied PPs within DoEs, the relative standard deviation of those PPs between large scale (LS) runs, and the monitored CQAs is given in Table 1.

### 2.2. Scope of IPM and Sampling Distribution of PPs

Due to the limited amount of quantitative analytical data of the CQAs before chromatography column 1, the starting distribution of each CQA at the first chromatography step was assumed to be normally distributed with mean and standard deviation estimated from measured CQA distribution of LS runs. From this starting point, the following unit operations chromatography column 1, 2, and 3 were modeled. The pool of chromatography column 3 was regarded as very similar to drug substance since no further clearance formation was expected at the ultrafiltration/diafiltration step.

For the MC workflow, we have to choose a realistic distribution of the large scale variation in process parameters in order to incorporate process-related variability. Results of the MC simulation are dependent on the sampling strategy for the process parameters at each simulation. Often pseudo-random numbers are replaced by quasi-random numbers or Latin hypercube sampling [10,11] for better overview of possible outcomes. However, for realistic risk assessment, we want our sampling to be representative for the process, therefore classical pseudo-random numbers were used for sampling. Existing variance of process parameters has been estimated from current large scale runs as listed in Table 1. We assumed a multivariate normal distribution for all process parameters centered at their mean (target of operation) and variance from large scale runs without any covariance between the process parameters. This is a suitable simplification since process parameters are controlled independently from each other. In general, this is not a prerequisite for the IPM and might be adapted for other processes, where additional information of potential correlation between the process parameters exists.

### 2.3. Impurity Clearance Models

Since it was aim of the IPM to model the final distribution of each of the four major CQAs (i.e., the specific concentration of each impurity) and the product in the drug substance, their reduction from load of chromatography column 1 until drug substance needs to be described mathematically. In order to estimate the specific CQA concentration after a unit operation (pool) using the specific load concentration of this CQA, specific clearances (SCs) were used (Equation (1)):

(1) $$\mathrm{Specific}\;\mathrm{Clearance}=\mathrm{SC}=\frac{c_{\mathrm{CQA},\mathrm{load}}}{c_{\mathrm{CQA},\mathrm{pool}}}$$

where $c_{\mathrm{CQA},\mathrm{load}}$ and $c_{\mathrm{CQA},\mathrm{pool}}$ is the specific CQA concentration, defined as the amount of impurity per amount of product, for load and pool, respectively.

For modeling the product a similar approach was chosen using step yields (SY) instead of SC (Equation (2)):

(2) $$\mathrm{StepYield}=\mathrm{SY}=\frac{p_{\mathrm{pool}}}{p_{\mathrm{load}}}$$

where $p_{\mathrm{pool}}$ and $p_{\mathrm{load}}$ are the amounts of product in pool and load, respectively.

Two major impacting sources specific clearances have been considered here: (i) Impact of potential critical process parameters, which have been purposefully selected in a risk assessment and (ii) specific amount of impurity load per column volume. Those types of models are described in more detail in the following two Section 2.3.1 and Section 2.3.2, respectively. In case it was not possible to find any PPs that significantly impact on the clearance, the mean clearance from LS was taken as a constant model (see Section 2.3.1 for details). We summarize all found models in Table 2.

#### 2.3.1. Clearance and Yield as a Function of Process Parameters (DoE Models)

As a general good practice in PV stage 1, after a purposeful selection of potential impacting process parameters, their impact on the SCs and the SY has been tested within DoEs. For reasons of simplicity, we will only show the modeling approach for SC in the following two sections and not for step yields, since both approaches are identical when exchanging SC with step yield. Experimental designs were chosen (see Table 1 for number of DoE runs and design) and linear models were established according to the form (Equation (3)):

(3) $$\mathrm{SC}={\;\mathrm{PP}*\beta }_{\mathrm{PP}}+\beta_{0}+\varepsilon$$

where SC is a (*n* × 1) vector of the measured specific clearances, PP is a (*n* × *p*) matrix of the process parameter settings of each DoE run, $\beta_{\mathrm{PP}}$ are the regression coefficients, and $\beta_{0}$ is the intercept. The process of selecting a subset of significant process parameters was accomplished by means of stepwise regression using multiple linear regression (MLR) package in inCyght (inCyght 2017.03, Exputec GmbH, Vienna, Austria). In this stepwise procedure, parameters showing a partial *p* value below 0.05 were allowed to enter the model starting with those parameters having the lowest partial *p* value. Partial *p* values of parameters can change as other parameters are included in a multivariate model. Therefore, after each time including a new parameter in the model, it is checked if *p* values of the existing parameters have increased and those parameters showing an *p* value larger than 0.1 will be excluded from the model. This including/excluding procedure was applied iteratively to achieve the optimal model, starting with the most significant parameter and was repeated as long as the model structure did not change any more. Thereby, ${\hat{\beta }}_{\mathrm{PP}}$ and ${\hat{\beta }}_{0}$ could be estimated. The herein obtained models and their respective statistical quality measures are summarized in Table S1 of the Supplementary materials.

A new prediction for SC ($\hat{\mathrm{SC}}$) for randomly selected set of process parameters of the *i*th MC simulation can be obtained by (Equation (4)):

(4) $$\hat{\mathrm{SC}}\left({\mathrm{PP}}^{\left(i\right)}\right)={\;\mathrm{PP}}^{\left(i\right)}{\;*\;\hat{\beta }}_{\mathrm{PP}}+{\hat{\beta }}_{0}$$

The prediction error of the mean model response was assumed to be normally distributed with: $N\left(\hat{\mathrm{SC}}\left({\mathrm{PP}}^{\left(i\right)}\right),\sigma {}^{2}{}_{\hat{\mathrm{SC}}\left({\mathrm{PP}}^{\left(i\right)}\right)}\right)$. Where $\sigma_{\hat{\mathrm{SC}}\left({\mathrm{PP}}^{\left(i\right)}\right)}$ was calculated using (Equation (5)):

(5) $$\sigma_{\hat{\mathrm{SC}}\left({\mathrm{PP}}^{\left(i\right)}\right)}=s_{\mathrm{SC}}*\sqrt{\frac{1}{n}+h_{i}}$$

with the leverage of the new data point: $h_{i}=\mathrm{diag}\left({\mathrm{PP}}^{\left(i\right)}{\left({\mathrm{PP}}^{\prime }\mathrm{PP}\right)}^{-1}{\mathrm{PP}}^{\left(i\right)}{}^{\prime }\right)$, the residual standard error: $s_{\mathrm{SC}}=\sqrt{\frac{\sum \left({\mathrm{SC}}_{i}-\hat{{\mathrm{SC}}_{i}}\right){}^{2}}{n-p-1}}$ if *p* are the number of parameters and n the number of observations. A random sample $\mathrm{rand}\left(N\left(\hat{\mathrm{SC}}\left({\mathrm{PP}}^{\left(i\right)}\right),\sigma {}^{2}{}_{\hat{\mathrm{SC}}\left({\mathrm{PP}}^{\left(i\right)}\right)}\right)\right)$, using MATLAB (MATLAB, The MathWorks Inc., R2015b, Natick, MA, USA) function randn, was taken from this prediction error distribution for each Monte Carlo simulation *i* and added to the mean prediction, obtaining the specific clearance impacted by PPs for each unit operation (Equation (6)):

(6) $$\tilde{\mathrm{SC}}\left({\mathrm{PP}}^{\left(i\right)}\right)=\mathrm{rand}\left(N\left(\hat{\mathrm{SC}}\left({\mathrm{PP}}^{\left(i\right)}\right),\sigma {}^{2}{}_{\hat{\mathrm{SC}}\left({\mathrm{PP}}^{\left(i\right)}\right)}\right)\right)$$

For responses where no further spiking models have been available, the specific CQA concentration of the pool of the *u*th unit operation was calculated to (Equation (7)):

(7) $$c_{\mathrm{CQA},\mathrm{pool},u}{}^{\left(i\right)}=\frac{c_{\mathrm{CQA},\mathrm{load},u}{}^{\left(i\right)}}{\tilde{\mathrm{SC}}\left({\mathrm{PP}}^{\left(i\right)}\right)}=\frac{c_{\mathrm{CQA},\mathrm{pool},u-1}{}^{\left(i\right)}}{\tilde{\mathrm{SC}}\left({\mathrm{PP}}^{\left(i\right)}\right)}$$

Note that here the concatenation of the unit operations occurs since the specific CQA concentration of the pool of unit operation u−1 is set equal to the load of unit operation u.

If no significant effects of any PP on an impurity clearance of a certain unit operation could be detected, a constant impurity clearance was assumed within the entire design space. This was modeled by the mean clearance of the LS runs and variance of the LS runs. In those cases, for each unit operation the specific clearance of the *i*th MC simulation reduces to (Equation (8)):

(8) $$\tilde{\mathrm{SC}}\left({\mathrm{PP}}^{\left(i\right)}\right)=\mathrm{rand}\left(N\left({\bar{\mathrm{SC}}}_{\mathrm{LS}},\sigma {}^{2}{}_{{\mathrm{SC}}_{\mathrm{LS}}}\right)\right)$$

where ${\bar{\mathrm{SC}}}_{\mathrm{LS}}$ and $\sigma {}^{2}{}_{{\mathrm{SC}}_{\mathrm{LS}}}$ is the mean SC and the variance from LS runs, respectively.

#### 2.3.2. Increased Clearance Due to Varied Spiking of Impurities

During process development and design, increased impurity levels were spiked on chromatographic preparative columns in order to show elevated clearance capacity. In more detail, during those spiking studies, it was shown that the impurity clearance increases with increasing impurity loading density ($\mathrm{ILD}=\frac{\mathrm{loaded}\;\mathrm{impurity}\;\mathrm{amount}}{\mathrm{column}\;\mathrm{volume}}$) up to the tested level. Additionally, the same relationship of increased impurity clearance at increased impurity loading densities was found for large scale runs, where the impurity loading varies for each run due to variation in fermentation and previous purification unit operations. Since the ILDs were not included within DoE approaches as an independent DoE factor, we followed a two-step approach to incorporate altered clearance at varying ILD.

**Figure 1:**
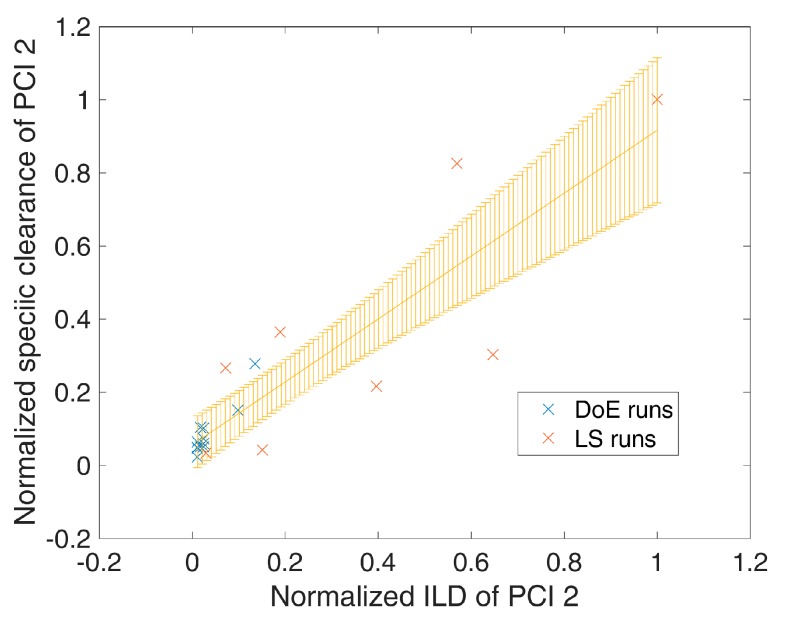
Exemplary plot for dependency of specific clearance (here of process-related impurity 2) against impurity loading density of process-related impurity 2 of DoE runs (blue) and large scale (LS) runs (red). Yellow error bars indicate the mean model prediction error. Normalization has been performed by division of the maximal value for each axis.

In the first step, linear regression on SC as a function of ILD was applied to identify significant correlations. Having such a regression model in place, for a specific ILD in the *i*th MC simulation an estimate for the SC could be obtained $\left(\tilde{\mathrm{SC}}\left({\mathrm{ILD}}^{\left(i\right)}\right)\right)$) (Equation (9)):

(9) $$\tilde{\mathrm{SC}}\left({\mathrm{ILD}}^{\left(i\right)}\right)=\mathrm{rand}\left(N\left(\hat{\mathrm{SC}}\left({\mathrm{ILD}}^{\left(i\right)}\right),\sigma^{2}{}_{\hat{\mathrm{SC}}\left({\mathrm{ILD}}^{\left(i\right)}\right)}\right)\right)$$

where $\hat{\mathrm{SC}}\left({\mathrm{ILD}}^{\left(i\right)}\right)$ is the mean predicted SC from the linear regression model at the specific ${\mathrm{ILD}}^{\left(i\right)}$ and $\sigma^{2}{}_{\hat{\mathrm{SC}}\left({\mathrm{ILD}}^{\left(i\right)}\right)}$ is the variance of the mean prediction, which can be obtained analogous to Equation (5). An example of such a spiking model is shown in Figure 1, where an increased loading density of process-related impurity 2 shows a significant (*p* = 7 × 10^−8^) increase in specific clearance of process-related impurity 2. Significant (*p*-value < 0.05 as well as R^2^ (explained variance) − Q^2^ (from leave one out cross validation) difference < 0.3) spiking models were selected for each response/unit operation and are summarized in Table 2 and Table S2 of the Supplementary Materials.

Hereafter in the second step, if significant spiking models were available, they were combined with the existing ones as a function of PPs as described in Section 2.3.1. Therefore, for each unit operation, we added the expected clearance increase due to increased ILD to the specific clearance of the *i*th Monte Carlo simulation impacted by PPs (Equation (10)):

(10) $${\tilde{\mathrm{SC}}}^{\left(i\right)}=\;\tilde{\mathrm{SC}}\left({\mathrm{PP}}^{\left(i\right)}\right)*\frac{\;\tilde{\mathrm{SC}}\left({\mathrm{ILD}}^{\left(i\right)}\right)}{\hat{\mathrm{SC}}\left(\bar{\mathrm{ILD}}\right)}$$

where $\hat{\mathrm{SC}}\left(\bar{\mathrm{ILD}}\right)$ is the SC under mean ILD from DoE runs. The ILD of the simulation *i* and the unit operation *u* can be calculated according to (Equation (11)):

(11) $${\mathrm{ILD}}_{u}^{\left(i\right)}=\frac{c_{\mathrm{load},u}^{\left(i\right)}*p_{\mathrm{load},u}^{\left(i\right)}}{\mathrm{CV}}=\frac{c_{\mathrm{pool},u-1}^{\left(i\right)}*p_{\mathrm{pool},u-1}^{\left(i\right)}}{\mathrm{CV}}$$

where $c_{\mathrm{load},u}^{\left(i\right)}$ is the specific concentration of the CQA at the *i*th simulation and the *u*th unit operation and $p_{\mathrm{load},u}^{\left(i\right)}$ is the product amount modeled by step yield of simulation *i* and unit operation *u*, CV is the column volume. Again, the load concentrations and amounts can be expressed by the respective pool concentrations of the previous unit operation (*u* − 1).

Since the impurity loading density was not included within DoE approaches on column steps as an independent DoE factor, we assume that varied impurities do not show interactive effects with other DoE factors (mainly process parameters) within normal operating variance. In order to estimate the risk that the simulation performance is biased by the spiking models and the risk of the above stated assumptions, the IPM was simulated without applying any spiking model. Those results are shown in Figures S1–S4 of the Supplementary materials, where we show that only for product-related impurity 1, process-related impurity 1, and process-related impurity 2, the out of specification chance increases by 0.1%, 0.7%, and 4.2%, respectively. Therefore, the above mentioned assumptions about spiking models and the connection to DoE models can be seen as a minor influence to the overall IPM prediction and valid simplification. Moreover, this can be regarded as a valid simplification since the assumed normal manufacturing variance which is used during IPM simulation of process parameters is well within the normal operating range (NOR, see standard deviation to NOR ratio in Table 1 is often below 30%) and therefore around 99% of the simulated batches are run within NOR. However, we want to note that one could even refine the IPM by including specific impurity concentrations in the load of chromatographic columns as an additional factor in DoE experiments to study that effect in combination with all other DoE factors.

## 3. Results

### 3.1. Monte Carlo Approach for Integrated Process Modeling

**Figure 2:**
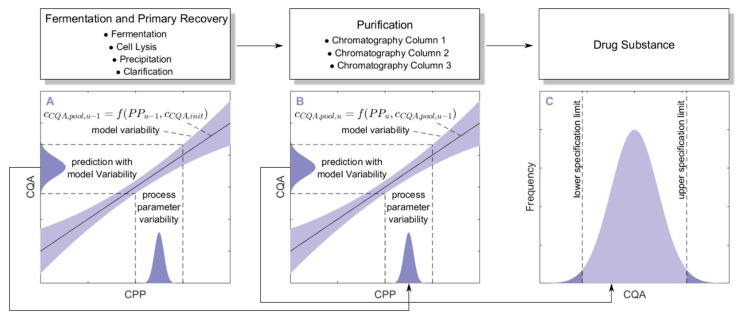
Schematic description of the integrated process model using a Monte Carlo approach: 1000 simulations are performed, each having a different set of process parameters (indicated as distribution on the *x*-axis of (**A**,**B**)) and initial specific CQA concentration $\left(c_{\mathrm{CQA},\mathrm{init}}\right)$. Multiple linear regression models describe the relationship between the $c_{\mathrm{CQA}}$ of the pool of unit operation *u* (**B**) and the PP of this unit operation as well as the pool concentration of the previous unit operation *u* − 1 (**A**). Thereby, models from multiple unit operations (**A**,**B**) are connected to predict the CQA distribution in the drug substance (**C**). Since 1000 simulations are performed, the CQA values form a distribution after each unit operation. The higher the model uncertainty, indicated by blue shaded area around the regression line, the wider the resulting CQA distribution. This ultimately propagates until drug substance, where the chance of out of specification events can be assessed.

The main idea behind the integrated process is to concatenate impurity clearance models of each unit operation together to predict the CQA distribution at each intermediate and at drug substance. To account for error propagation during this concatenation, we performed a Monte Carlo approach in four steps:

1. 1000 simulations were performed, each having a different set of PPs (${\mathrm{PP}}^{\left(i\right)}$) for the three modeled unit operations (chromatography column 1/2/3) and different initial specific CQA concentrations ($c^{\left(i\right)}{}_{\mathrm{CQA},\mathrm{init}}$) at the load of chromatography column 1, sampled from distributions which were estimated from LS runs. Also the variance in PPs was estimated from LS runs and is indicated by a schematic distribution on the *x*-axis in Figure 2A,B. Additional increase in simulations did not increase model accuracy and 1000 simulations are a common standard for Monte Carlo simulations [7]. A more detailed description of this step and a list of used process parameters are provided in Section 2.2.
2. For each unit operation, we modeled the specific clearance (SC) of each CQA as a function of the critical PPs and the ILD by multiple linear regression. Each model is associated with a prediction error, which is indicated by the blue shaded area around the found regression line Figure 2A,B. The ILD can be derived from $c_{\mathrm{CQA},\mathrm{load}}\;$ of each unit operation, which equals $c_{\mathrm{CQA},\mathrm{init}}$ for the first modeled unit operation and $c_{\mathrm{CQA},\mathrm{pool},u-1}$ for all subsequent modeled unit operations (*u*).
3. Since $c_{\mathrm{CQA},\mathrm{pool},u}$ can be calculated from SC and $c_{\mathrm{CQA},\mathrm{load},u}$, on the whole, $c_{\mathrm{CQA},\mathrm{pool},u}$ can be seen as a function of ${\mathrm{PP}}_{u}$ as well as $c_{\mathrm{CQA},\mathrm{init}}$ or $c_{\mathrm{CQA},\mathrm{pool},u-1}$, as indicated in the formula of Figure 2A,B, respectively. Thereby the model outputs from multiple unit operations can be stacked together, which is indicated by black arrows in Figure 2A, more thorough description of which models could be found on which CQA and unit operation is depicted in Section 2.3.
4. Since we performed 1000 simulations, each having different settings in process parameters, we obtained a distribution for the specific CQA concentration in the pool and finally in drug substance, indicated on the *y*-axis of Figure 2A,B and by the distribution in Figure 2C.

### 3.2. Validation of the IPM Using Observed CQA Distribution in Drug Substance

For model validation, the distribution of the predicted specific CQA concentrations at the pools of each unit operation and drug substance were compared to the measured CQA distribution of LS runs. The OOS chance for the IPM was calculated by simply counting the number of simulations that are above the upper specification limit and dividing by the number of simulations. For the calculation of the OOS chance using the 9 large scale runs, a normal distribution was fitted to the data.

**Figure 3:**
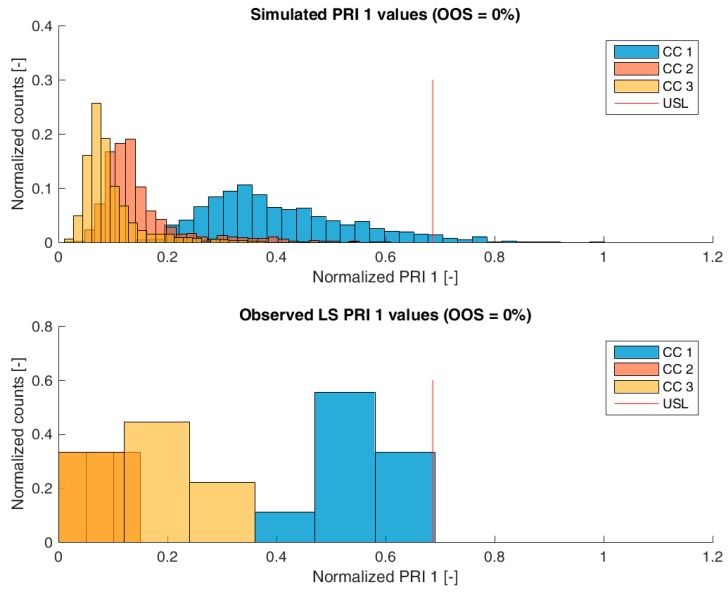
Comparison of simulated (**top**) product-related impurity 1 distribution and observed (**bottom**) product-related impurity 1 from LS after each column step. Normalization was performed by dividing by the maximum observed $c_{\mathrm{CQA}}$.

**Figure 4:**
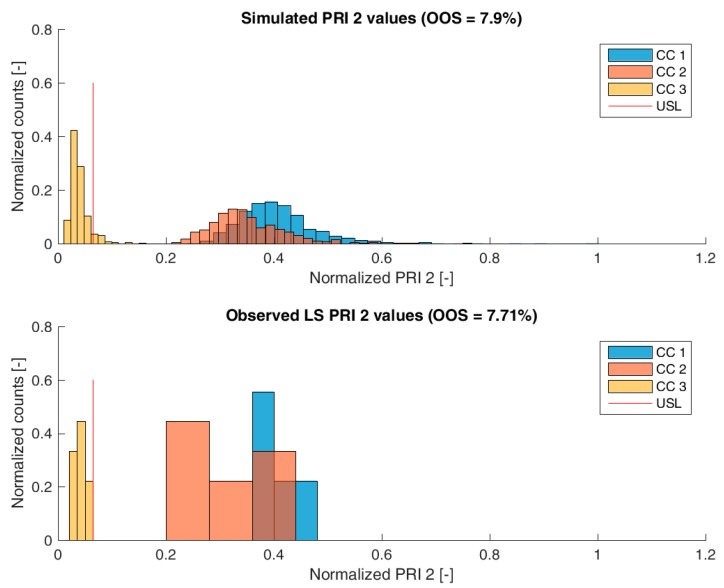
Comparison of simulated (**top**) product-related impurity 2 distribution and observed (**bottom**) product-related impurity 2 from LS after each column step. Normalization was performed by dividing by the maximum observed ${\;c}_{\mathrm{CQA}}$.

**Figure 5:**
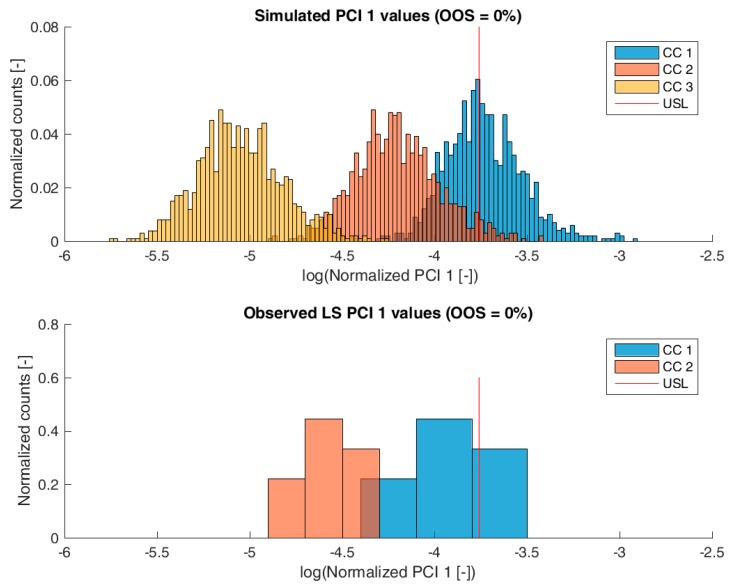
Comparison of simulated (**top**) process-related impurity 1 distribution and observed (**bottom**) process-related impurity 1 from LS after each column step. For chromatography column 3 pool, no process-related impurity 1 value was observed above LoQ, therefore, no histogram bar is plotted for the observed values at chromatography column 3 pool. Normalization was performed by dividing by the maximum observed $c_{\mathrm{CQA}}$.

**Figure 6:**
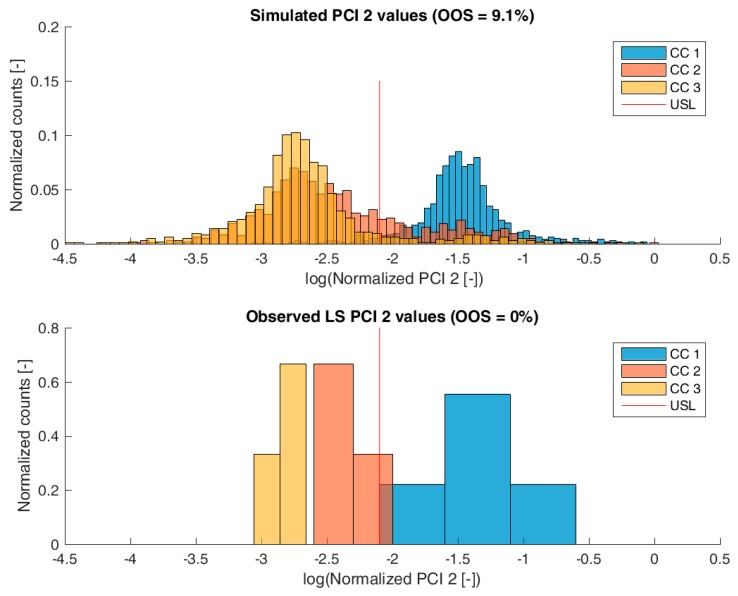
Comparison of simulated (**top**) process-related impurity 2 distribution and observed (**bottom**) process-related impurity 2 from LS after each column step. Normalization was performed by dividing by the maximum observed $c_{\mathrm{CQA}}$.

Figure 3, Figure 4, Figure 5 and Figure 6 show overlays of simulated and observed CQA distribution after each chromatography step for product-related impurity 1 and 2, as well as process-related impurity 1 and 2, respectively. For reasons of data security, all values have been normalized by the maximum observed or simulated CQA value. For the calculation of the observed distributions, all 9 LS runs have been used and have been plotted. CQA distribution after chromatography column 3 (yellow colored bar in Figure 3, Figure 4, Figure 5 and Figure 6) can be regarded as drug substance since no further purification has been shown to occur at the ultrafiltration/diafiltration step.

From visual inspection, the predicted distributions for each CQA nicely overlap with the observed distributions at each chromatography step. This is also reflected in good agreement of simulated and measured OOS probabilities at drug substance level, which are displayed in the title of each subfigure, except for process-related impurity 2. Also, the skewness of the measured CQA distribution is well described by the model (e.g., positive skewness of the product-related impurity 1 distribution at chromatography column 2 in Figure 3). Herein, we regard the model as valid for further investigations such as varying set-point conditions or accelerated variance of PPs.

For process-related impurity 2, the variance of the predicted specific CQA concentrations is larger than the observed variance, especially at chromatography column 3 level, as shown in Figure 6. However, the mean prediction at chromatography column 3 level is very close to the observed runs. The simulated OOS events of the IPM are 9.1% whereas only 0% when calculating from LS data. This gap in predicted versus observed OOS events might be caused by an different mean response of the scale down model at set-point conditions, which was used to conduct the experiments, an overlooked effect of a PP onto this CQA, an overlooked spiking model, or the gap is introduced by the selection of the current large scale runs which show a too low OOS chance. For the first two issues, power analysis for the insignificant models terms needs to identify if additional experiments need to be conducted to make sure that no critical effect has been overlooked [9]. Whereas, the latter possibility indicates a risk that was uncovered by the IPM and has luckily not been observed during LS runs. Herein, counter actions might be taken such as an increase of specific purification capacity in primary recovery.

For product-related impurity 2, the OOS chances for the IPM and the observed data are equally around 7%, as shown in Figure 4. Since for this CQA two statistical models as a function of PPs at chromatography column 1 and chromatography column 3 could be established (Table 2), parameter sensitivity analysis using the IPM can reveal optimization potential to increase process robustness for this CQA.

### 3.3. Impact of Accelerated Variation in Process Parameters on Drug Substance

Parameter sensitivity analysis (PSA) was performed to assess how a change in set-point or variance of controlled PPs influences OOS events at drug substance. PSA was conducted as follows: Each PP was varied individually regarding its mean and variance and resulting change in OOS events was measured. If interaction effects of parameters have been detected within DoE models, those parameters can be varied simultaneously to study this effect. However, this was not the case for any model established in this study. Moreover, since the model was built only on a segment of all unit operations, we are interested in how an altered performance of the fermentation and primary recovery—leading to an increased impurity burden at the load of chromatography column 1—will impact on drug substance. Therefore, the specific impurity concentration at the loading of chromatography column 1 was also varied in a parameter sensitivity analysis.

**Figure 7:**
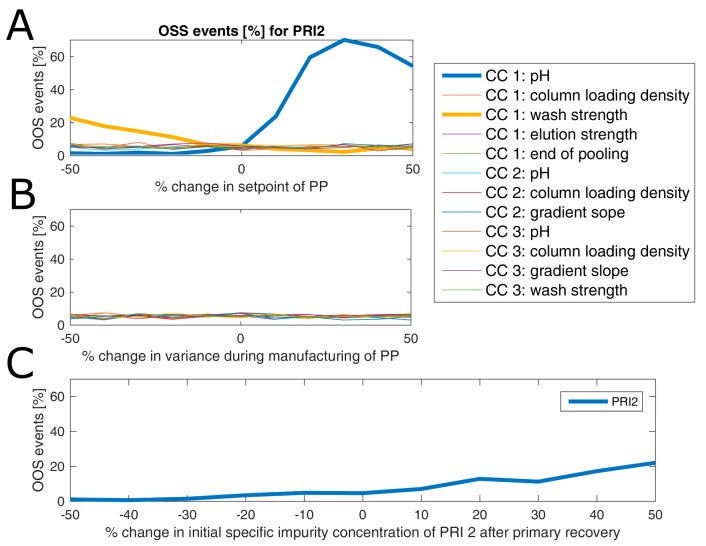
Estimated OOS event for product-related impurity 2 at drug substance as a function of change in set-point (**A**) and variance (**B**) of all PPs as well as a function of increased specific impurity concentration after primary recovery (**C**). Deviations in set-point of pH and salt concentration in wash of chromatography column 1 impact severely on OOS chance, which is not the case when variance in PPs increases by up to 50%. A change of specific product-related impurity 2 concentration at the primary recovery level will also increase OOS chances.

Results of an example of such an analysis are shown for product-related impurity 2 (Figure 7), where in panel A the change of OOS events as a function of change in percent of set-point settings of all process parameters is displayed. As can be seen from this subfigure, only a change in pH and wash strength of chromatography column 1 leads to a drastic change in OOS events. This is expected since both factors are part of the DoE model (see Table S1 of Supplementary materials). In more detail, both factors have a favorable direction in terms of reduction of OOS events (lowered pH and increased wash strength). For example, a reduction of the pH value by 10% of the set-point leads to a reduction of OOS events from 7% to around 3%. Interestingly, a change in variance of those two process parameters by ±50% does not impact the OOS events (Figure 7B). This sounds contradictory at first glance, however, since a variance increase to a certain extent will also drive a lot of simulations to the more favorable side (lowered pH and increased wash strength), the overall OOS chance remains similar to the initial estimate. This also emphasizes the well-known fact that optimization should be addressed via a change in the set-point rather than via reduction of variance, which is, in general, even harder to accomplish. A change in initial product-related impurity 2 burden after primary recovery propagates as well into drug substance, which can be explained by the fact that no spiking model could be established for this CQA at any unit operation, as shown in Figure 7C. In detail, a 10% reduction of the specific product-related impurity 2 concentration after primary recovery lowers the OOS events by another 3%. Therefore, it would be favorable to lower the pH of chromatography column 1 and reduce the impurity burden already after primary recovery using prior knowledge or build models that capture the interaction of fermentation and primary recovery parameters on this CQA. Thereby, OOS events could be lowered for product-related impurity 2 down to 1% or less. In order not to increase the OOS probability for another CQA by changing those two process parameters, one would need to also consider their impact on the residual CQAs. This is not shown here since we only wanted to introduce the methodology for a potential application of the IPM and due to reasons of simplicity.

## 4. Conclusions

Here we have shown how, by using an IPM, it was possible to demonstrate that sufficient process knowledge is available from process development to describe impurity clearance of process-related impurities 1 and 2, as well as product-related impurities 1 and 2. The distributions of simulated and observed CQAs are in good agreement to each other and make it possible to quantify the risk of not meeting product specifications under normal operating conditions, something which is often not possible due to limited large scale runs.

For product-related impurity 1 and process-related impurity 1, both the predicted OOS chance by the IPM as well as the observed OOS chance are numerically close to 0%. Herein, the process design can be validated in respect to those CQAs. In a first application of the IPM within a parameter sensitivity approach, it was possible to identify potential changes in process parameter set-points that will potentially decrease the chance of OOS events for product-related impurity 2 from 7% to 1%. For process-related impurity 2, the mean prediction of clearance within the IPM is similar to that obtained from LS measurements, however, the model predicts a 9.1% chance to be above drug substance specification, whereas current large scale data estimate 0% OOS chance. Since no statistical model could be established that might be used for optimization, process changes might be introduced. Here, IPM can be used within a model life-cycle approach as an enabler in change management. In case parts or entire unit operations are exchanged or included into an existing process design, the IPM can predict the mutual performance of this change in the context of existing clearance capacity. This can be achieved by replacement with statistical models of respective unit operations. Thereby, the overall performance of the changed process design can be assessed in terms of OOS events.

Furthermore, it should be emphasized that this model, in accordance with current opinion, is not finished in the traditional sense, but is expected to incorporate any future experiments and GMP runs for model refinement and application in further PV stages. Thereby, it is expected that new or insufficiently studied dependencies between PPs and CQAs can be incorporated as identified.

## Figures and Tables

**Table 1:** Available data sets, process parameters, and monitored critical quality attributes (CQAs) for each unit operation included in the integrated process model (IPM). CC is abbreviation for chromatography column, PCI stands for process-related impurities and PRI product-related impurities.

UO	Available Data Sets	PPs Varied in DoEs	Rel. Std. of PPs between LS [%] ^1^	Std/NOR [%] ^2^	Monitored CQAs
CC 1		pH [–]	1.61	46	PCI 1, PCI 2, PRI 1, PRI 2
		Column loading density [g/L]	12.05	50	
	9 manufacturing runs	Wash Strength [mM]	5.00	62	
	13 DoE runs with definitive screening design	Elution strength [mM]	5.00	44	
		End pooling [CV]	1.36	40	
CC 2	9 manufacturing runs	pH [–]	0.79	30	
	11 DoE runs using full factorial design	Column loading density [g/L]	4.84	20	
	1 spiking run with increased PRI 1 concentration in load	Gradient slope [% of Buffer]	5.00	-	
	1 spiking run with increased PCI 1 concentration in load				
CC 3		pH [–]	0.92	35	
	9 manufacturing runs	Column loading density [g/L]	12.78	30	
	9 DoE runs using definitive screening design	Gradient slope [% of Buffer]	5.00	-	
		Wash Strength [mM]	5.00	50	

^1^ Relative standard deviation to the set-point of the process parameters; ^2^ Ratio of one standard deviation to the normal operating range.

**Table 2:** Summary of the presence of models that describe the relationship of a CQA specific clearance factor as a function of PPs (indicated by “DoE”) or the impurity loading density of the respective CQA (“Spiking”) and the respective *p*-value of the regression. In cases where no significant function of PPs on a CQA clearance could be found, mean large scale clearance was assumed indicated by “LS clearance” in the table. CC is abbreviation for chromatography column, PCI stands for process-related impurities and PRI product-related impurities.

CQA/Unit Operation	CC 1	CC 2	CC 3
PRI 1	DoE	LS clearance + Spiking	DoE
	(linear, *p* = 0.09)	(*p* = 0.00)	(quadratic, *p* = 0.01)
PRI 2	DoE	LS clearance	LS clearance
	(linear, *p* = 0.01)		
PCI 1	DoE	LS clearance + Spiking	DoE
	(quadratic, *p* = 0.00)	(*p* = 0.04)	(quadratic, *p* = 0.00)
PCI 2	LS clearance + Spiking	LS clearance	LS clearance + Spiking
	(linear, *p* = 0.00)		(linear, *p* = 0.00)
Yield	DoE	LS clearance	DoE
	(linear, *p* = 0.00)		(quadratic, *p* = 0.00)

## Supplementary Materials

The following are available online at www.mdpi.com/2306-5354/4/4/86/s1, Figure S1: Comparison of simulated (**top**) product-related impurity 1 distribution and observed (**bottom**) product-related impurity 1 from LS after each column step, Figure S2: Comparison of simulated (**top**) product-related impurity 2 distribution and observed (**bottom**) product-related impurity 2 from LS after each column step, Figure S3: Comparison of simulated (**top**) process-related impurity 2 distribution and observed (**bottom**) process-related impurity 2 from LS after each column step, Figure S4: Comparison of simulated (**top**) process-related impurity 1 distribution and observed (**bottom**) process-related impurity 1 from LS after each column step, Table S1: Overview of found models based on DoE data, Table S2: Overview of models showing a correlation between specific CQA clearances and CQA load density.
