# Supplementary material for: Integrated Process Modeling—A Process Validation Life Cycle Companion
Source: Bioengineering (Basel). 2017 Oct 17;4(4):86. doi: 10.3390/bioengineering4040086 (PMC5746753; doi:10.3390/bioengineering4040086)
Supplement: Supplementary file 1 [file bioengineering-04-00086-s001.pdf]

Table S1: Overview of found models based on DoE data. CC is abbreviation for chromatography column, PCI stands for process related impurities and PRI product related impurities.

	Response	alpha	Model	R^2	Q^2	ΔRQ	P	Parameters
CC 1	Yield	0.05	Linear	0.88	0.74	0.14	0.000	(+) End pooling,<br>(+) Elution strength,<br>(-) pH
	Specific PRI 1 clearance	0.05	Linear	0.26	0.05	0.20	0.092	(+) pH,
	Specific PRI 2 clearance	0.05	Linear	0.77	0.50	0.27	0.006	(-) pH,<br>(+), Wash strength,<br>(+) column loading density
	Specific DNA clearance	-	-	-	-	-	-	-
	Specific PCI 1 clearance	0.05	Quadratic	0.91	0.62	0.30	0.003	(+) End pooling,<br>(-) End pooling^2,<br>(+) Column loading density,<br>(+) Wash strength,<br>(-) pH
	Specific PCI 2 clearance	-	-	-	-	-	-	-
CC 2	Yield	-	-	-	-	-	-	-
	Specific PRI 1 clearance	-	-	-	-	-	-	-
	Specific PRI 2 clearance	-	-	-	-	-	-	-
	Specific DNA clearance	-	-	-	-	-	-	-
	Specific PCI 1 clearance	-	-	-	-	-	-	-
	Specific PCI 2 clearance	-	-	-	-	-	-	-
CC 3	Yield	0.05	Quadratic	0.91	0.80	0.11	0.000	(-) Column loading density^2,<br>(+) Column loading density
	Specific PRI 1 clearance	0.05	Quadratic	1.00	0.91	0.09	0.009	(-) pH^2,<br>(+) pH,<br>(-) Wash strength,<br>(+) Wash strength^2,<br>(-) Column loading density^2,<br>(+) Column loading density
	Specific PRI 2 clearance	-	-	-	-	-	-	-
	Specific DNA clearance	-	-	-	-	-	-	-
	Specific PCI 1 clearance	0.05	Quadratic	0.99	0.98	0.02	0.000	(+) Column loading density,<br>(-) Column loading density^2,<br>(+) Gradient slope
	Specific PCI 2 clearance	-	-	-	-	-	-	-

Table S2: Overview of models showing a correlation between specific CQA clearances and CQA load density. CC is abbreviation for chromatography column, PCI stands for process related impurities and PRI product related impurities.

	Response	alpha	model	R^2	Q^2	ΔRQ	P	Parameters
CC 1	Specific PRI 1 clearance	-	-	-	-	-	-	-
	Specific PRI 2 clearance	-	-	-	-	-	-	-
	Specific DNA clearance	-	-	-	-	-	-	-
	Specific PCI 1 clearance	-	-	-	-	-	-	-
	Specific PCI 2 clearance	0.05	Linear	0.78	0.68	0.10	0.000	Load PCI 2 amount per CV
C	Specific PRI 1 clearance[1]	0.05	Linear	0.66	0.42	0.24	0.000	Load PRI 1 amount per CV

	Specific PRI 2 clearance	-	-	-	-	-	-	-
	Specific DNA clearance	-	-	-	-	-	-	-
	Specific PCI 1 clearance[2]	0.05	Linear	0.54	0.36	0.18	0.000	Load PCI 1 amount per CV
	Specific PCI 2 clearance	-	-	-	-	-	-	-
CC 3	Specific PRI 1 clearance	-	-	-	-	-	-	-
	Specific PRI 2 clearance	-	-	-	-	-	-	-
	Specific DNA clearance	-	-	-	-	-	-	-
	Specific PCI 1 clearance	-	-	-	-	-	-	-
	Specific PCI 2 clearance	0.05	Linear	0.63	0.37	0.26	0.002	Load PCI 2 amount per CV

[1] PRI 1 spiking experiments were used to establish this model

[2] PCI 1 depletion experiments were used to establish this model

IPM Simulation without Spiking Models

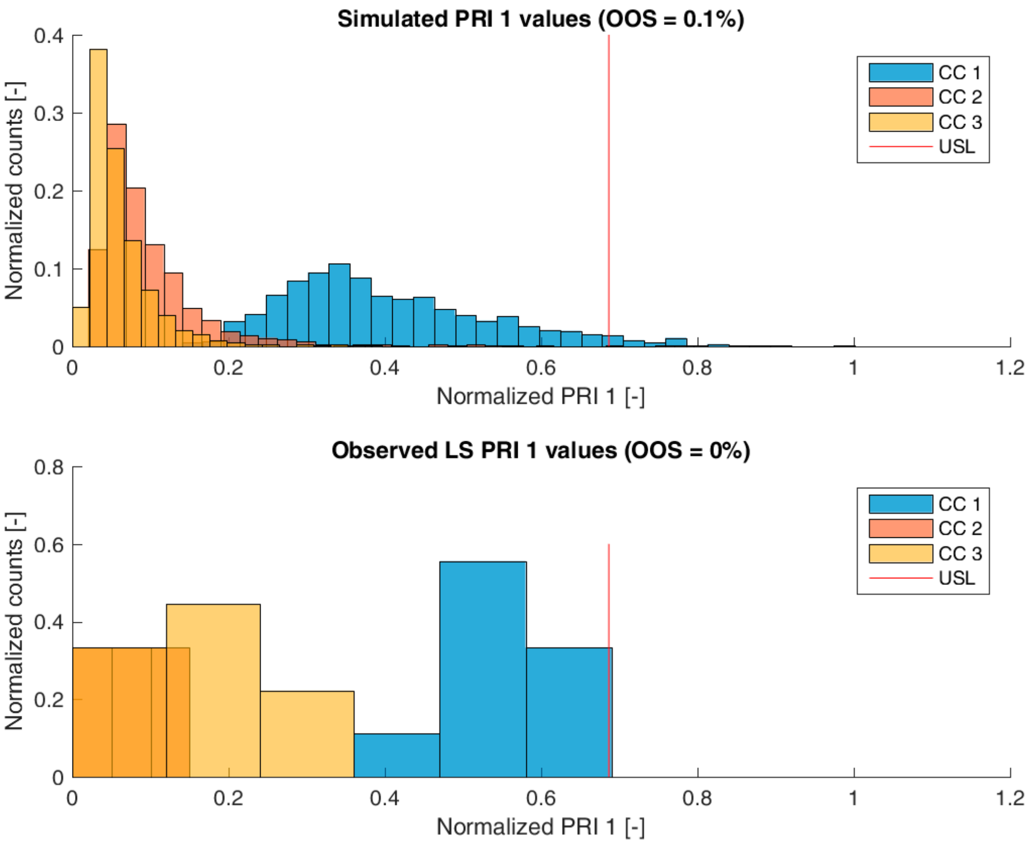

Figure S1: Comparison of simulated (top) product related impurity 1 distribution and observed (bottom) product related impurity 1 from LS after each column step. Normalization was performed by diving by the maximum observed c_{CQA}. Simulation was performed without taking any spiking model into account.

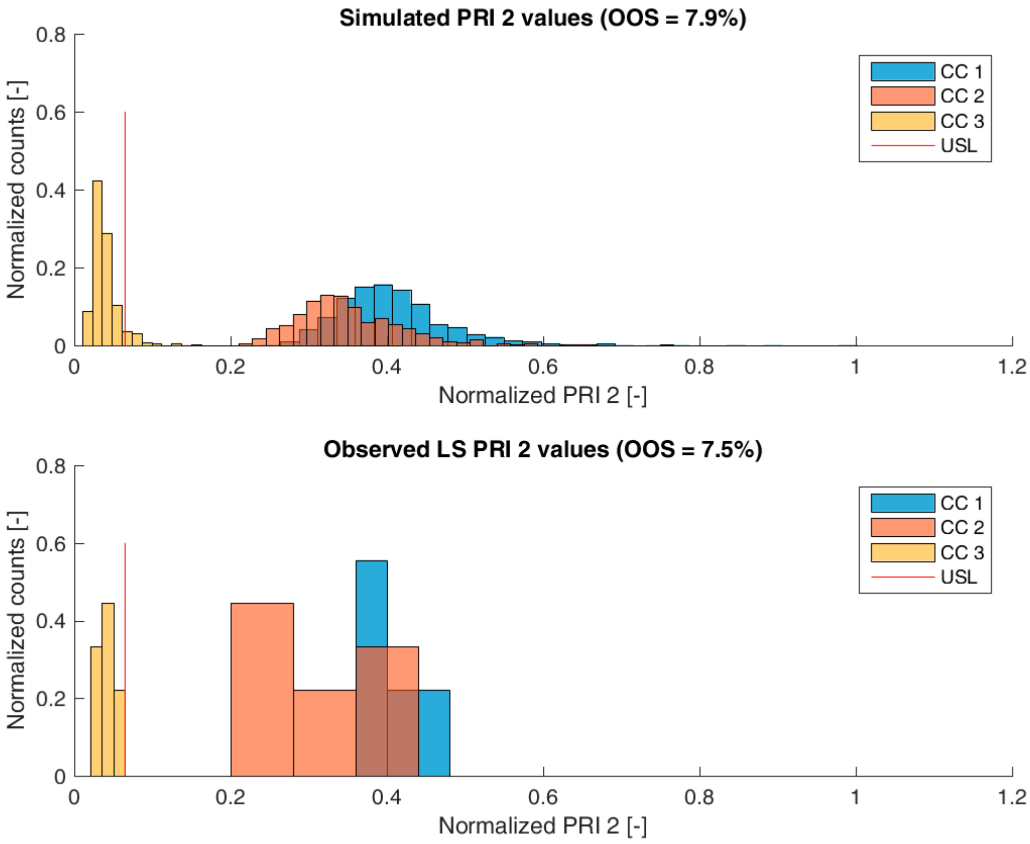

Figure S2: Comparison of simulated (top) product related impurity 2 distribution and observed (bottom) product related impurity 2 from LS after each column step. Normalization was performed by diving by the maximum observed c_{CQA}. Simulation was performed without taking any spiking model into account.

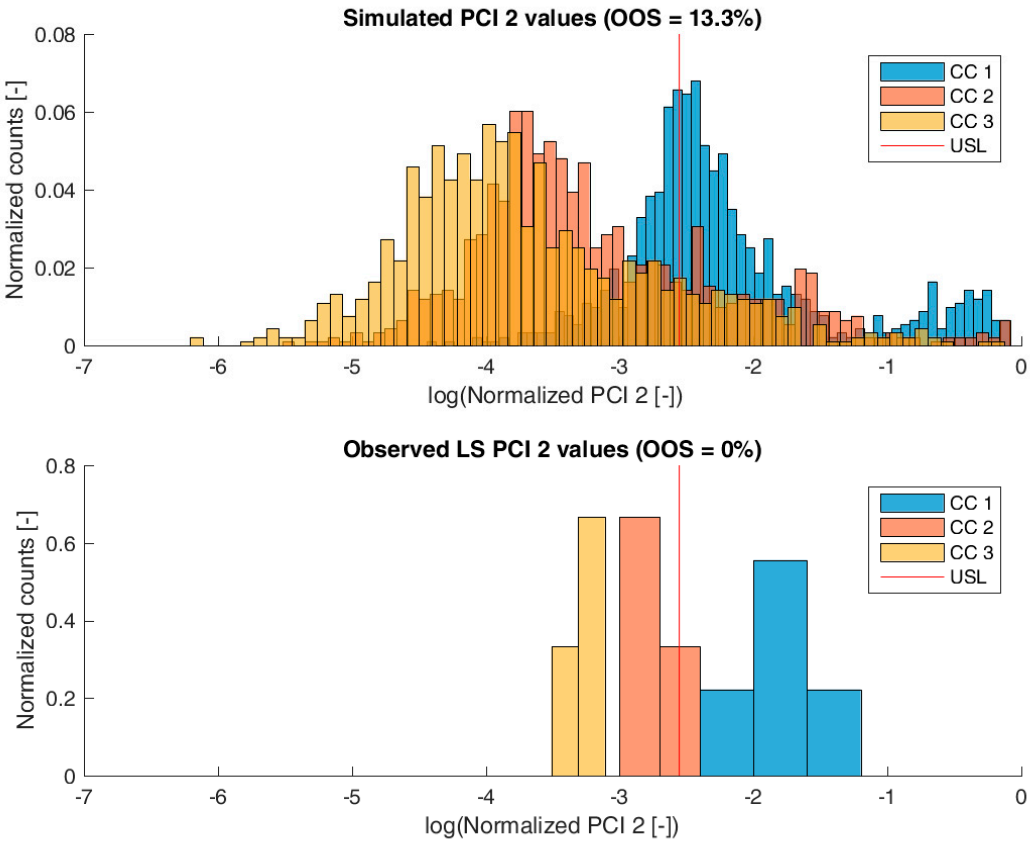

Figure S3: Comparison of simulated (top) process related impurity 2 distribution and observed (bottom) process related impurity 2 from LS after each column step. Normalization was performed by diving by the maximum observed c_{CQA}. Simulation was performed without taking any spiking model into account.

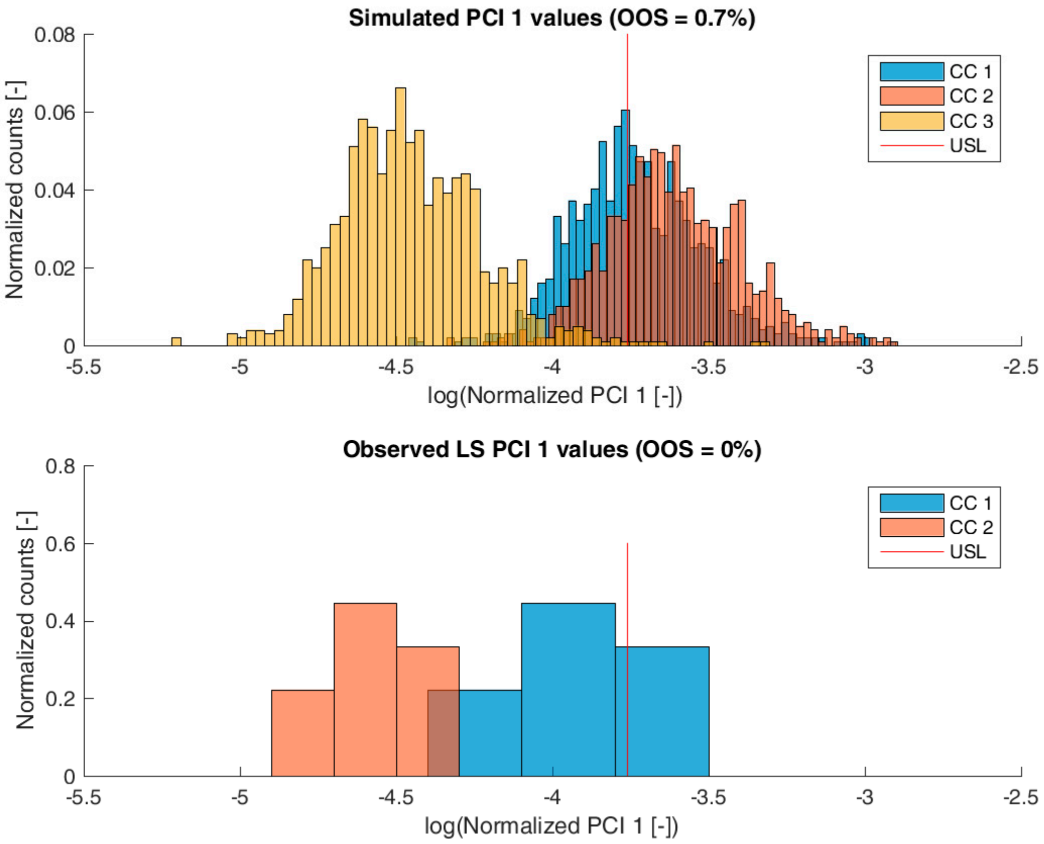

Figure S4: Comparison of simulated (top) process related impurity 1 distribution and observed (bottom) process related impurity 1 from LS after each column step. For CC 3 pool, no process related impurity 1 value was observed above LoQ, therefore, no histogram bar is plotted for the observed values at CC 3 pool. Normalization was performed by diving by the maximum observed c_{CQA}. Simulation was performed without taking any spiking model into account.
